# Prevalence and differences in the co-administration of drugs known to interact: an analysis of three distinct and large populations

**DOI:** 10.1186/s12916-024-03384-1

**Published:** 2024-04-19

**Authors:** Jon Sánchez-Valle, Rion Brattig Correia, Marta Camacho-Artacho, Rosalba Lepore, Mauro M. Mattos, Luis M. Rocha, Alfonso Valencia

**Affiliations:** 1 Life Sciences Department, Barcelona Supercomputing Center, 08034 Barcelona, Spain; 2 Instituto Gulbenkian de Ciência, 2780-156 Street, Oeiras Portugal; 3 Agencia Española de Medicamentos y Productos Sanitarios, Madrid, Spain; 4 Department of Biomedicine, Basel University Hospital and University of Basel, CH-4031 Basel, Switzerland; 5 Universidade Regional de Blumenau, Blumenau, 89030-903 Brazil; 6 Department of Systems Science and Industrial Engineering, Binghamton University, Binghamton, 13902 USA; 7 ICREA, 08010 Barcelona, Spain

**Keywords:** Drug–drug interactions, Polypharmacy, Multimorbidity, Electronic health records

## Abstract

**Background:**

The co-administration of drugs known to interact greatly impacts morbidity, mortality, and health economics. This study aims to examine the drug–drug interaction (DDI) phenomenon with a large-scale longitudinal analysis of age and gender differences found in drug administration data from three distinct healthcare systems.

**Methods:**

This study analyzes drug administrations from population-wide electronic health records in Blumenau (Brazil; 133 K individuals), Catalonia (Spain; 5.5 M individuals), and Indianapolis (USA; 264 K individuals). The stratified prevalences of DDI for multiple severity levels per patient gender and age at the time of administration are computed, and null models are used to estimate the expected impact of polypharmacy on DDI prevalence. Finally, to study actionable strategies to reduce DDI prevalence, alternative polypharmacy regimens using drugs with fewer known interactions are simulated.

**Results:**

A large prevalence of co-administration of drugs known to interact is found in all populations, affecting 12.51%, 12.12%, and 10.06% of individuals in Blumenau, Indianapolis, and Catalonia, respectively. Despite very different healthcare systems and drug availability, the increasing prevalence of DDI as patients age is very similar across all three populations and is not explained solely by higher co-administration rates in the elderly. In general, the prevalence of DDI is significantly higher in women — with the exception of men over 50 years old in Indianapolis. Finally, we show that using proton pump inhibitor alternatives to omeprazole (the drug involved in more co-administrations in Catalonia and Blumenau), the proportion of patients that are administered known DDI can be reduced by up to 21% in both Blumenau and Catalonia and 2% in Indianapolis.

**Conclusions:**

DDI administration has a high incidence in society, regardless of geographic, population, and healthcare management differences. Although DDI prevalence increases with age, our analysis points to a complex phenomenon that is much more prevalent than expected, suggesting comorbidities as key drivers of the increase. Furthermore, the gender differences observed in most age groups across populations are concerning in regard to gender equity in healthcare. Finally, our study exemplifies how electronic health records’ analysis can lead to actionable interventions that significantly reduce the administration of known DDI and its associated human and economic costs.

**Supplementary Information:**

The online version contains supplementary material available at 10.1186/s12916-024-03384-1.

## Background

Adverse drug reactions (ADR) are noxious or unintended effects related to drug administration. ADRs are a major public health problem due to their impact on morbidity, mortality, and health economics [1, 2]. The co-administration of drugs may cause ADRs from a drug–drug interaction (DDI), defined as the effect one drug has on another at the pharmacokinetic or pharmacodynamic level. ADRs have been associated with 4.2 to 8.4% of all hospital admissions [2, 3], and of these, about 51% are related to DDIs [2], while other studies estimate a median DDI prevalence rate of hospital admissions around 1% to almost 2% [4, 5]. These numbers increase with polypharmacy, which has been described to have doubled from 1995 to 2010, also increasing the percentage of individuals taking DDIs from 5.8 to 13.1% [6]. The risk of ADR-related hospital admission goes up from fivefold for patients treated with more than three drugs to ninefold for those treated with more than 10 drugs [2]. As the population ages, the risk of suffering from two or more chronic conditions at the same time (known as multimorbidity) increases. This increase is different for women and men, both in terms of prevalence and the specific diseases that co-occur [7, 8]. As a consequence, the instances of polypharmacy and the prevalence of DDI also increase [9, 10], reaching a prevalence of 46% in the elderly, where 10% of them take severe interactions [11]. Regarding the differences in the prevalence of DDI according to age, a higher prevalence has been described in men during childhood [12], followed by a higher prevalence in adult women under 80 years of age and a higher prevalence in men over 80 [13]. Potentially, this rise occurs differently due to differences in the co-occurrence of diseases between the two genders [10, 14–16].

Factors in addition to age and gender, such as errors and lack of information in ambulatory care [17, 18] and the number of physicians prescribing drugs [19], are also known to increase the risk of DDIs. Often, physicians are unaware of the complete list of the drugs their patients are taking [17]. To counter this, computerized health information systems (HIS) such as electronic health records (EHR), drug interaction software, and decision support systems have been developed to screen for DDIs proactively and alert physicians and pharmacists [20] even though reports of preventable ADR-related hospital admissions vary widely, from 24 to 52% [21, 22] to 77 to 92% of all ADR-related hospital admissions [2, 23], HIS attempt to lower these rates. However, HIS alone are insufficient to prevent prescription errors, as physicians may dismiss alerts [24] as they lack context and clinical relevance. Indeed, 55 to 98% of the DDI alerts are overridden [25]. To solve the problem, algorithms that take into consideration patients’ context from EHR have been developed, reducing the number of alerts by more than 50% [26]. Together, these distinct factors paint a picture of a complex DDI phenomenon with worrying direct consequences for patients and health systems. For instance, our previous analysis revealed that DDIs likely account for a significant financial burden to public health, reaching 2 dollars per capita in a city in Brazil during 18 months—extrapolated to an expenditure of $565 M for the country in the same period [10].

Despite the problem’s relevance, most studies have focused on specific populations with limited sample sizes. In addition, most of these studies focus on the analysis of narrow age ranges—primarily on patients over 65 or pediatric patients—making it difficult to understand the alterations that occur throughout life. Furthermore, each study follows different methodological procedures, highlighting the need for joint analyses of different populations. To better untangle the factors involved in the global DDI phenomenon, we analyze administration patterns retrieved from EHR from three large populations with distinct public and private healthcare systems: Blumenau (Brazil; pop. 338,876), Catalonia (Spain; pop. 7.6 million), and Indianapolis (USA; pop. 876,682). We study demographic variables, such as age and gender, as well as drugs involved in DDIs in all three populations in detail. In addition, we evaluate the role of polypharmacy and co-administration by building a statistical null model that shuffles drug labels while accounting for cohort-specific drug availability. Finally, we demonstrate the population-level impact of individual DDIs by simulating the administration of drug alternatives to omeprazole, a commonly prescribed proton pump inhibitor with several known and avoidable interactions.

## Methods

### Data—Blumenau

Blumenau is a city in Brazil. Drugs reported in the *Pronto* HIS are available via medical prescription only, free of charge, and administered to citizens of Blumenau. Via *Pronto*, doctors prescribe medications by selecting drugs and dosages, and pharmacists dispense them by selecting quantity. This allows us to estimate the length of drug administration in days. We note that patients are not required to retrieve drugs from the public system. They can buy prescribed medications from private pharmacies at their own expense without such transactions being recorded in *Pronto*. Drug names originally in Portuguese have been translated to English, disambiguated, and matched to their IDs in DrugBank, an open-source drug database that contains DDI information. Medications with multiple drug compounds have been split into their constituent drugs. Administered substances not matched in DrugBank were discarded. These commercial EHRs contain 18 months (Jan 2014–Jun 2015) of anonymized drug administration and patient demographics retrieved from *Pronto*. It is the same data used in Correia et al. [10] except for the removal of ophthalmological drugs, topical drugs, and vaccines from the analysis. In total, we analyze 140 unique DrugBank IDs dispensed to 133*,*047 patients. The study was approved by Indiana University’s Institutional Review Board.

### Data—Catalonia

Catalonia is an autonomous community of Spain. The data includes 11 years (Jan 2008–Dec 2018) of anonymized drug billing data, disease diagnoses (International Code of Diseases, 10th version (ICD-10)), and patient demographics provided by the Catalan Health Institute (CHI), and extracted from the SIDIAP (Information System for Research in Primary Care). The CHI manages primary healthcare teams that serve 74% of the Catalan population. All the CHI care professionals have used the same computerized clinical history program (e-CAP) in all visits (medical and nursing) since 2005 to register the mentioned demographic information, prescriptions, disease diagnoses, and laboratory tests [27]. The data was thus gathered for administrative purposes. Drugs are identified by their Anatomical Therapeutic Chemical (ATC) classification, which contains five levels of detail. We use the finest detail level—chemical substance—and remove topical drugs. For comparison, we map ATC codes to DrugBank IDs. Importantly, we note that (a) a drug can map to more than one ATC code when it has different routes of administration or therapeutic uses and (b) some ATC codes represent combined drugs. For simplicity, we aggregate all ATC code billing that matches a DrugBank ID and split combined drugs into their constituent drugs. Drug billing is given at a monthly resolution. Only patients born before January 2007 were included in the study. In total, we analyzed 814 unique DrugBank IDs administered to 5,555,924 patients. The study was approved by the Jordi Gol University Institute for Research Primary Healthcare ethics committee. This manuscript has not been prepared in collaboration with this registry(s) and therefore does not necessarily reflect their opinions or points of view. The quality and accuracy are the sole responsibility of the author of the manuscript.

### Data—Indianapolis

Indianapolis is a city in the USA. Two years (Jan 2017–Dec 2018) of commercial EHR data were purchased from the Regenstrief Institute. This nonprofit organization provides research access to the Indiana Network for Patient Care. This health information exchange system contains 13 billion data elements from more than 100 hospital systems and thousands of providers across the state, with most of the data being from the city of Indianapolis. The data we obtained under an agreement contains anonymized disease diagnoses (ICD-10), patient demographics, drug quantity, and treatment duration for all three care levels. Unlike the other populations, drugs in this dataset could have been administered as prescribed by primary care physicians or in a hospital setting. Treatment duration allows us to estimate the length of administration in days. Similarly to the Blumenau data, we disambiguate individual medication names, match them to DrugBank IDs, and split medications with multiple drug compounds into their constituent drugs. After removing ophthalmological drugs, topical drugs, and vaccines, we analyzed 1228 unique DrugBank IDs dispensed to 264,607 patients. The study was approved by Indiana University’s Institutional Review Board.

### Drug–drug interactions

**Fig. 1:**
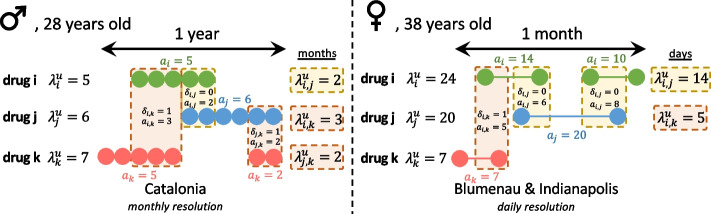
Diagram of co-administration and interaction computation for Catalonia, Blumenau, and Indianapolis. Two hypothetical patient-drug dispensing timelines with three drugs (*i*, *j*, and *k*) are represented. In Catalonia (left), two drugs (*i,j*) are assumed to be co-administered if they were dispensed and billed during the same month. In Blumenau and Indianapolis (right), two drugs are assumed to be co-administered if they were dispensed for an administration period with an overlap of at least 1 day. Drug administration lengths (in days for Blumenau and Indianapolis, and months for Catalonia) are shown for each dispensation. The three possible pairwise comparisons (*i,j*), (*i,k*), and (*j,k*) between the dispensed drugs are shown with their co-administration overlap marked with backgrounds in either orange (not known DDI) or red (known DDI). Note: medications dispensed together are not necessarily taken together, they may be distributed throughout the day to avoid certain interactions

To ensure all DDIs found from the earliest dispensation dates in our study to the most recent, we use the 2011 version of DrugBank as our drug interaction reference. Since using different time windows may affect the prevalence detected (a more extended study period increases the probability of detecting drug co-administrations and DDI), we have analyzed this prevalence using the same time window (18 months, the smallest available for Blumenau) and the complete study periods (2 years for Indianapolis and 11 years for Catalonia). Following the notation proposed in Correia et al. [10], we denote patients by *u* ∈ *U*, and drugs by *i,j* ∈ *D*, where *U*_*i*_ ∈ *U* represents the subset of patients dispensed drug *i*, and *D*^*u*^ ⊆ *D* is the subset of drugs dispensed to patient *u*. Since patients can be administered a drug *i* multiple times during the study period, we denote the set of distinct administration intervals *a*^*i,u*^_*n*_ (in days or months) of drug *i* to patient *u* as ${A}_{i}^{u}\equiv \{{a}^{i,u}\}$. The total number of administrations and time units a patient *u* is administered a drug *i* are denoted by ${\alpha }_{i}^{u}=|{A}_{i}^{u}|$ and ${\lambda }_{i}^{u}=\sum {a}^{i,u}$. For Blumenau and Indianapolis, we are able to compute drug administration length in days. For Catalonia, however, we only have monthly drug billing data; therefore, in this case, ${\alpha }_{i}^{u}={\lambda }_{i}^{u}$ denotes the number of months drug *i* was administered to patient *u*. We assume dispensed drugs were administered for the entire prescribed length. Similarly, the number of distinct co-administration periods of two drugs (*i* and *j*) to patient *u* and the length of co-administration are denoted by ${\alpha }_{i,j}^{u}$ and ${\lambda }_{i,j}^{u}$, respectively (see Fig. 1). For each observed DDI, we manually retrieve a severity score (major, moderate, and minor) from drugs.com [28], a website containing drug information, including DDI descriptions. From these values, we compute other quantities and sets per patient *u*, drug *i*, or drug pair (*i,j*).

To characterize the *conditional likelihood of a drug pair* (*i,j*) in the population (${\gamma }_{i,j}^{\psi }$), we divided the number of patients who administered the drug pair concomitantly, $|{U}_{i,j}^{\psi }|$, by the number of patients who administered one of the drugs in the pair, to obtain the probability that patients who administered drug *i* also co-administered drug pair (*i,j*). Values of ${\gamma }_{i,j}^{\psi }$ closer to 1 indicate that drug *j* is usually co-administered with drug *i* in the population, or vice-versa for ${\gamma }_{j,i}^{\psi }$, as this measure is not symmetrical $({\gamma }_{i,j}^{\psi }\ne {\gamma }_{j,i}^{\psi })$.

Since ${\gamma }_{i,j}^{\psi }$ does not differentiate if drugs *i* and *j* are concomitantly administered for a short or long period of time, and we assume that the length of DDI administration is relevant for ADRs, we also characterize the length of co-administration of drug pairs to a patient *u* by calculating the strength of co-administration $({\tau }_{i,j}^{u}$). The strength is calculated by dividing the duration of the co-administration by the duration of separate administration of the drugs (Additional file 1: Supplementary Material), where ${\tau }_{i,j}^{u}\in [\mathrm{0,1}]$. This measure of *normalized co-administration length* per patient differentiates between drug pairs with complete temporal overlap, $({\tau }_{i,j}^{u}\to 1)$, and with a small temporal overlap $({\tau }_{i,j}^{u}\to 0)$. Its mean value for the cohort of patients who administered drug pair (*i,j*) concomitantly yields a measure of *strength of co-administration* of the pair in the population (Additional file 1: supplementary material [29–31]). This proximity measure defines a weighted, undirected graph ${T}^{\psi }$ [10] on set *D* with edges, ${\tau }_{i,j}^{\psi }\in [\mathrm{0,1}]$, that relate drugs in the patient population according to the strength of co-administration (as inferred by normalized co-administration length).

Co-administrations of interacting drugs can be represented as a graph (${T}^{\phi }$). Graph ${T}^{\phi }$ synthesizes the multivariate DDI phenomenon in a given population as a network. To test the significance of the observed DDIs in the population, we calculate Fisher’s exact tests on the number of patients affected by each DDI, $|{U}_{i,j}^{\phi }|$, and the Bonferroni adjusted *p*-value based on the total number of DDI found in each population. Interacting drug pairs with a false discovery rate (FDR) ≤ 0.05 are considered significant and further analyzed.

For each population, we calculate the *prevalence of co-administration* (*PC*) as the number of patients who co-administered at least two drugs divided by the total number of patients. Similarly, we calculate the *prevalence of interaction* (*PI*) as the proportion of patients in the population who are administered at least one DDI.

### Gender prevalence

The relative risk of co-administration (*RRC*) for women is computed as the prevalence of co-administration in women divided by the prevalence of co-administration in men. The relative risk for men is calculated inversely. Similarly, we also compute the relative risk of interaction (*RRI*) for women as the prevalence of DDI in women divided by the prevalence of DDI in men (and inversely for women). Additionally, Fisher’s exact tests are used to calculate the significance of the various measures.

### Age prevalence

To evaluate the effect of patient age on the DDI phenomenon, we bin patients into 5-year age groups (or age cohorts) to compute an age-dependent prevalence of co-administration and DDI. In other words, the prevalence of co-administration of drugs in each age range is calculated as the percentage of patients in that age range who are co-administered drugs. The prevalence of co-administration of drugs in an age range is calculated as the percentage of patients in that age range who are co-administered drugs. Similarly, the prevalence of interactions in each age group is calculated as the percentage of patients exposed to DDI in the corresponding age group. Both calculations are repeated separately for women and men. This allows us to compute relative risks constrained by age ranges, gender, and drug pairs. Note that due to the temporal nature of our study, patient age is calculated based on their date of birth and the date of the drug event. This means that individual patients may be accounted for in multiple independent age ranges.

### Drug–drug interaction network

To synthesize, depict, and analyze the DDI phenomenon captured by the EHR data, we build a DDI network for each population where nodes represent drugs and edges denote an observed and significant drug interaction in the population (Fisher’s exact test, FDR ≤ 0.05). Each population network is defined by graph ${T}^{\phi }$, further refined such that edge width is proportional to the strength of DDI, while edge color represents the gender-specific relative risk for women in darker red and men in darker blue. Further, node size denotes the probability of patients who administered drug *i* to be exposed to a DDI associated with that drug $P\left({U}_{i}^{\phi }\right)$ and is computed as the number of patients exposed to a DDI involving drug i divided by the number of patients taking drug *i*. An interactive application allowing users to filter results and explore the associated network is available at http://disease-perception.bsc.es/ddinteract/.

### Null model

The null model captures the expected increase in DDI prevalence with age, given observed polypharmacy and patient demographics within each age group. We assume a random administration of drugs to patients in a specific age group, therefore maintaining the same number of unique drugs dispensed and co-administered for each randomly drawn patient. Specifically, we randomly draw patients from each age group. Then, for each patient, we randomly “dispense” drugs drawn from a set of drugs observed to be dispensed to patients in the same age group. In other words, in the null model, patients “administer” the same number of drugs as in the observed real population, but the drugs are randomly selected from the set of drugs observed to be prescribed for that age group. The expected prevalence of DDIs is then calculated for each age group, as was done with the observed data. Then, odds ratios are calculated to investigate the prevalence disparity between the actual data and the null model by Fisher’s exact tests.

Furthermore, the null model also uses the same number of “co-administered” drug pairs (*i*,*j*) as observed in the real data, with the co-administered drugs *j* also drawn randomly from the set of “administered” drugs to user *u* in the null model. As in the original analysis, these random drug pairs are subsequently checked for DDI status in DrugBank. We repeat this random sampling process 100 times and compute all derived prevalence measures, as done with the original data.

### Removal of omeprazole-associated interactions

Since omeprazole is known to be over-prescribed and has one of the largest numbers of interactions observed in our study (see Additional file 1: Table S1 and Table S2), we simulate the replacement of omeprazole with alternative PPI in observed DDI cases. We use the ATC drug classification system that describes chemical subgroups containing drugs that could, in principle, be interchanged for treating the same disease to identify alternatives. Thus, as proof of concept, we focus on the PPI subgroup: omeprazole, pantoprazole, esomeprazole, lansoprazole, and rabeprazole. We then replace, in each situation, omeprazole with the alternative that avoids interactions with other drugs and recalculate the previously described prevalence measures.

## Results

### Population comparison

In order to best compare the three populations, we first analyze the initial 18 months (the smallest temporal window available, for Blumenau) of administrations in each population. This is necessary as longer study periods increase the chances of observing co-administrations and DDIs and could bias our conclusions. We find that 140, 814, and 1228 unique drugs were dispensed respectively in Blumenau, Catalonia, and Indianapolis, with 106 drugs common to all three populations (Additional file 1: Fig. S1A). Considering the complete set of drugs administered in each population, they present a very similar prevalence of co-administration (*PC*) with the largest for Blumenau (76.99%), followed by Catalonia (75.78%) and Indianapolis (74.16%). This prevalence increases to 89.83% for Catalonia and 75.53% for Indianapolis when we analyze all available data (11 and 2 years, respectively; see Additional file 1: Table S3). The three populations also observe a similar prevalence of drug interaction (*PI*), with the largest again for Blumenau (12.51%), but closely followed by Indianapolis (12.12%) and then Catalonia (10.06%). The prevalence of co-administration (Additional file 1: Table S4) and DDI (Additional file 1: Table S5) are significantly higher in Blumenau compared to Catalonia and Indianapolis. Interestingly, while the prevalence of overall drug co-administration is significantly lower in Indianapolis compared to Catalonia (OR = 0.979), DDI prevalence is higher for Indianapolis (OR = 1.206). The DDI prevalence increases to 20.36% for Catalonia and 13.04% for Indianapolis when we analyze all available data (11 and 2 years, respectively; see Additional file 1: Table S3). Further leveraging all available data we show that the DDI phenomenon is more similar between Catalonia and Blumenau (0.52, Spearman correlation, see Additional file 1: Fig. S1C), in comparison to Indianapolis and either Catalonia (0.3) or Blumenau (0.27).

Given the common set of 106 drugs, we observe 149 known DDI pairs co-administered in all three populations (Additional file 1: Fig. S1B). As shown in Additional file 1: Table S6, digoxin is the drug most often administered to patients in conjunction with its interacting drugs in Blumenau and Indianapolis (Additional file 1: Table S6). For instance, in the three populations, from all patients who were administered digoxin, 47–60% of them also co-administered furosemide. Conversely, for all patients who were administered furosemide, only 4–12% also co-administered digoxin. This DDI also has one of the largest observed strength of drug interaction (Additional file 1: Table S6), which shows that it tends to be administered for long periods of time, increasing the risk of hospitalization due to digoxin intoxication [30].

In addition, 10 out of the 12 shared DDI (Additional file 1: Table S6) are related to cardiovascular disorders. Only two pairs make up the exception: valproic acid–carbamazepine and haloperidol–lithium cation. The former are anticonvulsants usually prescribed to treat seizure and bipolar disorders and given in combination to boost mood stabilization when monotherapy using either drug fails [32]. The latter are antipsychotic drugs used to treat schizophrenia and bipolar disorder and combined to provide modest, statistically significant benefits in the treatment of schizoaffective disorder [33]. Even if both drugs are not frequently given together, our results denote a stronger association of Lithium cation with haloperidol rather than the other way around, potentially due to the smaller effectiveness of lithium alone compared with other neuroleptics.

Finally, we find that half of the shared DDIs pose major health risks, such as hyperkalemia and kidney failure (spironolactone–losartan), increased risk of bleeding (warfarin–amiodarone), and excess mortality (digoxin–amiodarone) [29]. Indeed, the digoxin-amiodarone interaction is among the DDIs most frequently associated with hospital admissions and visits [4].

### Gender prevalence comparison

We observe only a slightly higher but significant relative risk of co-administration for women in the three populations: Blumenau (*RRC* = 1*.*07), Indianapolis (*RRC* = 1*.*06), Catalonia (*RRC*= 1*.*05) (see Table 1). This relative risk increases substantially when focused on interacting drugs, especially in Blumenau (*RRI* = 1*.*54), but is also high in Catalonia (*RRI* = 1*.*25) and present in Indianapolis (*RRI* = 1*.*12). Drug combinations that cause moderate interactions, which should be used only under special circumstances because of their clinically significant outcomes, are the most co-administered in all three populations and drive the differences between genders (see Table 1).

**Table 1:** Relative risk for women (*RRW*) of drug co-administration (*RRC*) and interactions (RRI); the latter is also computed for types of interactions as per drugs.com (minor, moderate, and major). The percentage of patients of each gender (*M*, man; *W*, woman) for each case is also shown. Values shown for all three populations during the first 18 months of the study. Asterisks denote statistically significant differences based on Fisher’s exact test results

	Blumenau		Catalonia		Indianapolis	
	RRW	% W, M	RRW	% W, M	RRW	% W, M
Co-administration (RRC^W^)	1.07*	(79.08%, 74.06%)	1.05*	(77.39%, 73.88%)	1.06*	(77.88%, 71.82%)
Interaction (RRI^W^)	1.54*	(14.64%, 9.49%)	1.25*	(11.08%, 8.84%)	1.12*	(12.69%, 11.36%)
Minor interaction	0.81*	(0.36%, 0.44%)	1.27*	(0.26%, 0.2%)	1.02	(1.13%, 1.1%)
Moderate interaction	1.59*	(11.3%, 7.1%)	1.4*	(8.31%, 5.93%)	1.12*	(10.67%, 9.49%)
Major interaction	1.53*	(6.25%, 4.07%)	1.08*	(3.17%, 2.95%)	1.02	(4.87%, 4.76%)

### Age prevalence comparison

**Fig. 2:**
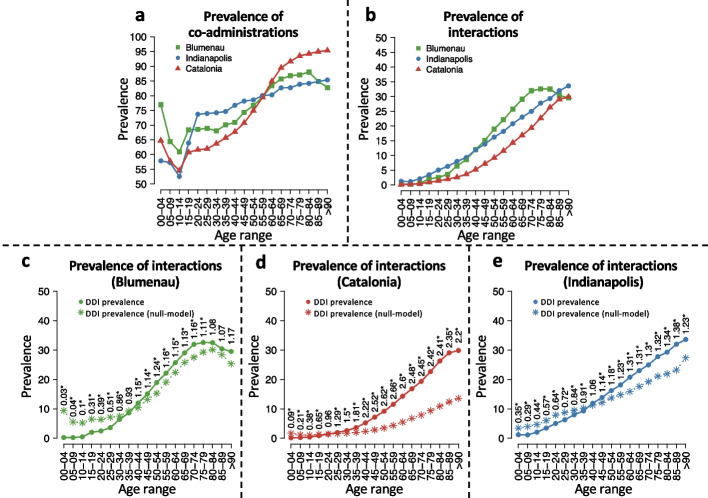
Prevalence of co-administration and interaction by age during the first 18 months of the studies. Green, red, and blue lines denote measurements for Blumenau, Catalonia, and Indianapolis, respectively. **a** Prevalence of co-administration of drugs. **b** Prevalence of co-administration of drugs known to interact. **c**–**e** Prevalence of interactions against the respective null model in **c** Blumenau, **d** Catalonia, and **e** Indianapolis. Circles denote the values obtained with the real data, while the asterisks denote the values obtained using the null model. The associated relative risk is shown above the points. Asterisks denote significant differences (Fisher’s exact test)

To analyze the effect of patient aging on the prevalence of drug co-administration and DDIs, we divide patients into age intervals of 5 years, based on their age at the time of administration (see the “Methods” section). As a well-known polypharmacy phenomenon, the prevalence of co-administration increases with age in all three populations as depicted in Fig. 2a; Additional file 1: Fig. S2 depicts the proportions of patients per number of drugs simultaneously co-administered. It is noteworthy that there is a drop in *PC* in the 10–14 age range for all three populations. Patients in the 15–59-year-old range in Catalonia have the lowest *PC*, although the largest *PC* is also observed in Catalonia for patients older than 59. Conversely, it is in Indianapolis that the largest *PC* is observed for 20–59-year-old patients.

The prevalence of a DDI increases with age from less than 0.2% of patients in the 0–4 year range, to up to 33.6% of patients over 90 years old (see Additional file 1: Fig. 2b). After the age of 75, *PI* is at least 20% for all three populations, and over 32% for Blumenau. Interestingly, all three populations display monotonically increasing *PI* with age (except for the oldest two age groups in Blumenau), despite their widely different cultures, available medications, and healthcare systems. Despite this, there are some noteworthy differences among the three populations as well. For instance, Indianapolis has the highest *PI* in patients age 0–39 as well as those older than 85. Blumenau, on the other hand, has the highest *PI* for patients age 40–84, being Catalonia the one with the lowest *PI* across all age groups, even though its patients age 60–90 have the highest *PC* (compare Fig. 2a, b).

Our previous study [10] indicated that regression models do not explain well the relationship between co-administrations and interactions, even when including all variables available in data as co-variates and for arbitrary regression complexity (also no evidence of a nonlinear relationship between co-administrations and interactions). Therefore, we build a statistical null model, marked in Fig. 2c–e with asterisks, which yields the expected DDI co-administration for each age range if patients were prescribed (age-specific) drugs at random, to evaluate what proportion of the observed increasing *PI* with age in all three populations (Fig. 2b) is explained by the also increasing *PC*. Random prescription of drugs is of course oblivious to know DDI information, so one would expect actual prescription—given available information about DDI—to result in lower prevalence than the null model. Indeed, this is observed for younger age groups, as the actual *PI* is lower than that of the null model with random drug administration. Thus, younger patients present a lower-than-random prevalence of DDIs for their rate of drug co-administration. However, and much to our surprise, for patients over 20 years of age in Catalonia and over 40 years of age in Blumenau and Indianapolis, the actual *PI* significantly surpasses what would be expected by chance: a worse-than-random chance of administering DDIs. This means that the higher prevalence of drug interactions faced by older age groups cannot be explained solely by increasing polypharmacy, pointing to comorbidity relationships as possibly responsible for this higher-than-expected prevalence. Indeed, previous studies have highlighted that the main risk factors for adverse drug events are multimorbidity and polypharmacy [34].

### Gender prevalence by age comparison

**Fig. 3:**
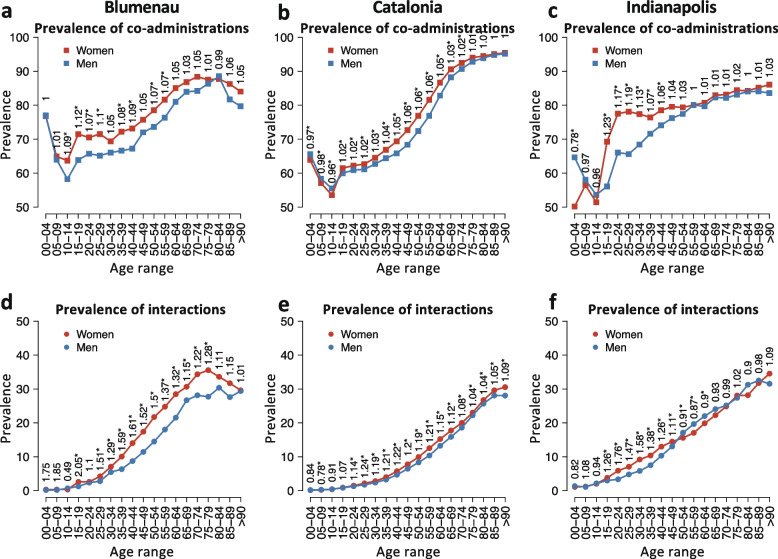
**a**–**c** Prevalence of drug co-administrations and (d-f) interactions by age and gender for Blumenau, Catalonia, and Indianapolis in the first 18 months of administration. Red and blue colors denote the prevalence in women and men, respectively. Relative risks of co-administration and interaction for women per age group are displayed above the points. Asterisks denote significant differences (Fisher’s exact test)

To study the role of gender in the observed age-associated prevalence of co-administration and DDI during the first 18 months of data in all three populations, we also analyze men and women separately. Figure 3a–c shows that women consistently have a higher prevalence of drug co-administration throughout their lifetime in all three populations, when compared to men. Nonetheless, this relative risk is typically small, being significant in almost all age ranges in Catalonia and only in specific age ranges in the cases of Indianapolis and Blumenau (15–29 years old). Overall, in Catalonia, we observe the smallest *RRC* across all ages, with greater gender imbalance in co-administration observed in Blumenau and Indianapolis showing across most age groups in the former, and greater imbalance for women only in age group 15–44 in the latter.

The cross-population comparison of the prevalence of gender-related drug interaction across age groups reveals some similarities as well as more nuanced differences. *RRI* is higher for women in Blumenau and Catalonia in almost all age ranges, with the exception of younger age groups (10–14 in Blumenau and 0–14 in Catalonia as shown in Fig. 3d, e). In contrast, in Indianapolis, men present a higher prevalence of DDI in the 50–89 age range, significantly so for patients aged 50–64, as seen in Fig. 3f. Nonetheless, the relative risk of interaction reaches higher values for women than for men in all three populations. In Catalonia, which presents the most gender-balanced scenario across age groups, women aged 25–59 face a significantly higher prevalence of DDI in comparison to men near or above 20% (*RRI* ≥ 1*.*19). Interestingly, when we analyze all 11 years of data for Catalonia, the relative risk for younger women (15 to 59 years) is also above 20% with *RRI* ≥ 1*.*2 (see Additional file 1: Fig. S3e). In fact, when analyzing all 11 years’ worth of data, the largest relative risk of DDI for women is observed in the 15–29 age range, which correlates with higher ethinylestradiol administrations in the years 2012-2018 (Additional file 1: Fig. S4a and Fig. S5d-e).

In Indianapolis, women aged 15–44 face a prevalence of interaction at least 26% higher than men (*RRI* ≥ 1*.*26), peaking at 20–24 (*RRI* = 1*.*76). In Blumenau, women aged 25–64 face a prevalence of interaction in comparison to men near or above 30% (*RRI* ≥ 1*.*29), reaching a peak at 40–44 (*RRI* = 1*.*61). In summary, across the three populations, women between 15 and 49 face a substantially higher DDI prevalence than men—the largest relative risk is observed in Blumenau for women aged 15–19 (*RRI* = 2*.*05). When compared to the null model, we note that the worst-than-random prevalence of interactions happens earlier for Catalan women (15–19 age range) than for men (20–24) (Additional file 1: Fig. S6). For Blumenau and Indianapolis, there is no gender difference when comparing to the null model.

Naturally, DDIs can cause different levels of adverse events, from mild headaches to patient hospitalization due to liver damage complications. Thus, we study the gender-associated differences based on the severity of the DDI, by tallying the number of women and men in each age range while accounting for minor, moderate, and major DDIs. DDI severity is extracted from drugs.com [28] (see the “Methods” section). Results are shown in Additional file 1: Fig. S7 and Fig. S8 and indicate that moderate DDIs are the most common with increasing patient age. In addition, in Indianapolis, the shift in gender-associated prevalence is largely explained by moderate DDIs, more common in women 15–49 years old and in men over 50 (Additional file 1: Fig. S7j). An interesting pattern of elevated prevalence in major DDIs in older men is also present in both Catalonia and Indianapolis, but not Blumenau. In Catalonia, men have a higher prevalence of major DDIs in the ages 50–84 (Additional file 1: Fig. S7g), while in Indianapolis men have a higher prevalence of major DDIs in ages 45–84 (Additional file 1: Fig. S7k). Since drugs.com is tailored to a US audience, drugs administered in other countries and their associated interactions may not be included in the site. The differences in the prevalence of these DDI are very similar in the three populations, being higher for women in Blumenau, and for men in Catalonia and Indianapolis.

### Drug interaction networks

**Fig. 4:**
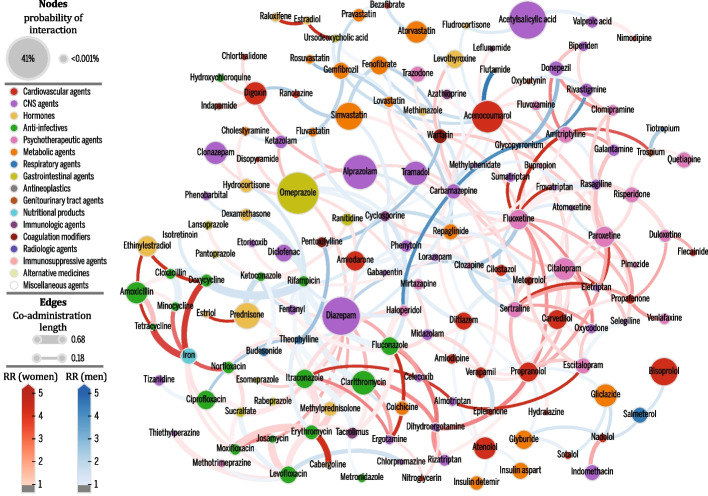
Catalonia DDI network. Nodes denote drugs involved in at least one co-administration known to be a DDI. Only nodes connected via edges with a strength of interaction larger than 0.18 are shown for clarity. Node color represents the highest level of primary action class, as retrieved from drugs.com. Node sizes are proportional to the probability of patients being affected by a DDI involving the drug (*P*(*U*_*i*_^Φ^)). Edge weights denote the strength of interaction (co-administration length). Edge colors denote relative risk (RR) for women (red) or men (blue). Color intensity for relative risks varies in [1, 5]; that is, values are clipped at 5 for clarity

To better characterize the DDI phenomenon in each of the three populations, we build drug–drug interaction networks shown in Fig. 4 and Additional file 1: Fig. S9, Fig. S10, and Fig. S11.

Nodes are colored based on their drugs.com category and sized based on the probability that patients prescribed the drug will experience a DDI. Edge width represents the strength of drug interaction and edge color denotes the gender-associated relative risk of a DDI, with red (blue) denoting higher prevalence in women (men). An interactive version of these networks can be explored at http://disease-perception.bsc.es/ddinteract/.

These networks help us not only visualize which drugs are most involved in interactions but also identify pairs with the same gender-associated differences (edge color) in all populations. For instance, considering the 149 DDIs common to all three populations, 56% are associated with increased prevalence in the same gender (56 DDIs for women, 27 for men). In addition, the network representation facilitates inferences for specific drugs or categories. For instance, drug interactions involving fluconazole, contraceptives, or benzodiazepines are more prevalent in women, while most interactions involving anticoagulants (such as warfarin interacting with phenytoin, prednisone, amiodarone, etc.) are more prevalent in men.

Conversely, there are drug pairs where the gender-associated difference is reversed in at least one population, with Blumenau presenting the highest discordance: 27 pairs. Interestingly, 11 of these 27 discordant interactions are major DDIs, including the concomitant use of ASA (anticoagulant) and ibuprofen (anti-inflammatory), a combination that reduces the effectiveness of aspirin in preventing stroke and increases the risk of developing gastrointestinal ulcers (Additional file 1: Table S7).

### Drug interactions driving gender-associated differences

**Fig. 5:**
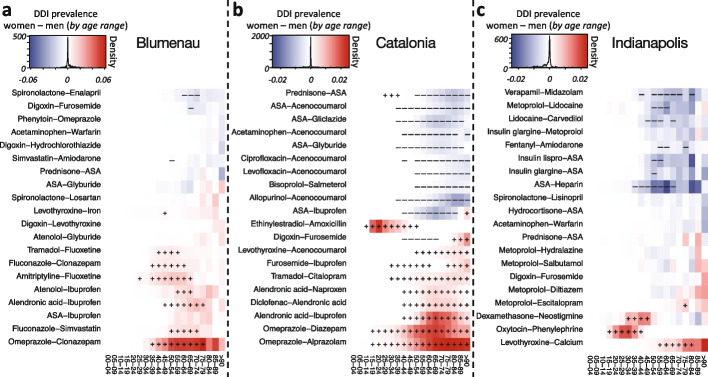
Top 20 drug interactions with the highest difference between DDI prevalence in women and men. Colors denote a higher prevalence of interactions in women (red) and men (blue). Markers (+ and −) denote significantly higher prevalence of DDI administrations in the respective gender after correcting for multiple testing (FDR ≤ 0.05). Note the color scale is different across populations, as the maximum and minimum differences in DDI prevalence are different between populations

Among the shared drug interactions in all three populations (149), we observe a strong association between omeprazole and both clonazepam and diazepam for women in Blumenau and Catalonia (see red cells in Fig. 5a, b), but not in Indianapolis (see Additional file 1: Fig. S12). This is particularly supported by the over-administration of omeprazole in the two populations (Additional file 1: Table S2). Similarly, the prevalence of co-administering alendronic acid—used to treat osteoporosis—and nonsteroidal anti-inflammatories is higher for women, paired with diclofenac in Catalonia and ibuprofen in both Blumenau and Catalonia. This DDI may result in an increased risk for stomach and intestine irritation. The co-administration of ethinylestradiol (contraceptive) and amoxicillin (antibiotic) is significantly high in all three populations. This DDI may result in reduced contraceptive effectiveness, thus increasing the risk of unwanted pregnancy. Interestingly, the major interaction between ASA and ibuprofen previously observed to be associated with a higher prevalence in women in Blumenau [10] is conversely associated with a lower prevalence in women in the other two populations (Fig. 5 and Additional file 1: Fig. S12), suggesting a particularity of the Blumenau healthcare system. This result points to the existence of cultural or social factors that play a role in this gender-associated difference. Another interesting DDI case that may point to social or cultural factors is the drug pair lidocaine–carvedilol, that only presents a higher prevalence in men in Indianapolis (Additional file 1: Fig. S12).

Looking further at the DDIs with high gender- and age-associated prevalence in each population (Fig. 5), we notice in Blumenau a significantly higher prevalence of co-administration of fluoxetine (major depression treatment) with tramadol (opioid analgesic) or amitriptyline (tricyclic antidepressant) in women. In Catalonia, for men over 40 years old, the prevalence of co-administration of anticoagulants such as ASA and acenocoumarol either with each other or with anti-diabetic drugs (gliclazide and glyburide), allopurinol (gout treatment), prednisone (glucocorticoid anti-inflammatory), or antibiotics (ciprofloxacin and levofloxacin) is significantly higher (Fig. 5b). Lastly, in Indianapolis young women present a significantly higher prevalence of co-administration of oxytocin, used to induce labor, and phenylephrine, used to increase blood pressure (Fig. 5c). For women older than 55 in Indianapolis, there is also a significantly higher prevalence of co-administration of levothyroxine, used to treat hypothyroidism, with calcium, which can change the absorption levels of levothyroxine. Conversely, we also found drug pairs with an increased prevalence in men. For instance, the combination of two anticoagulants, ASA and heparin; verapamil (a calcium channel blocker) and midazolam (benzodiazepine); lidocaine (a local anesthetic) with metoprolol and carvedilol (a beta-blocking agent); and anti-diabetic drugs, such as insulin lispro and insulin glargine, with ASA and metoprolol, a beta1 receptor blocker used to treat high blood pressure that can increase the risk of hypoglycemia. Specific interacting pairs can be visualized at http://disease-perception.bsc.es/ddinteract/.

### Alternative drug treatments to avoid DDIs

While the observed DDIs involving omeprazole and either clonazepam or diazepam are mostly irrelevant in Indianapolis (administered to 256 and 135 patients, respectively), they are the most co-administered drug pairs in Blumenau (5,076, 998) and Catalonia (47,811, 253,473). Here, we analyze the preferential co-administration of omeprazole over alternative proton-pump inhibitors (PPI) that have no known drug interaction with benzodiazepines in Catalonia (see the “Methods” section). Catalonia presents a significant preferential co-administration of omeprazole with diazepam or clonazepam, as compared to other PPI as a group (i.e., esomeprazole, pantoprazole, rabeprazole, and lansoprazole) (OR = 17.6 and 12.2, respectively) or individually (Additional file 1: Table S8). Conversely, in Indianapolis, there is a significant preferential administration of alternative PPI in combination with diazepam or clonazepam (OR = 38.3 and 13.5). Importantly, alternative PPI are available for administration in Catalonia, which is not the case for the public healthcare system of Blumenau where they can only be purchased from private pharmacies. Indeed, 12 of the 16 (75%) drugs associated with omeprazole interactions can be avoided using an alternative PPI.

Based on this observation, we first simulate for Catalonia the population-level effect of removing the omeprazole-associated interactions from the overall DDI prevalence. In this simulation, we replace omeprazole with currently available alternative PPI and recalculate the DDI prevalence. We find that administering alternative PPI reduces the overall levels of DDI in Catalonia by 23.28% in women and 20.09% in men (Additional file 1: Fig. S13b). The majority of these avoidable omeprazole interactions are generating moderate adverse effects (Additional file 1: Fig. S14b,e), which affect 18.85% (12.31%) of men (women) and can be avoided in 34.82% and 32.9% of the patients. For Indianapolis, the same simulation only reduces overall DDI levels by 2.55% in men and 2.56% in women (Additional file 1: Fig. S13c). Though no omeprazole substitutes are available free of charge in Blumenau, we followed the same simulation procedure using the alternatives available in Catalonia. Interestingly, the percentages of preventable interactions are almost identical to those in Catalonia, 23.19% for women and 19.51% for men (Additional file 1: Fig. S13a).

## Discussion

This is the first study to analyze DDI administration patterns in three large populations with distinct healthcare systems. We analyzed the medication administration records of nearly six million patients from up to 11 years of data. Despite different study periods and data resolutions for each population, similar patterns were revealed. The prevalence of drug co-administrations and interactions by age are both similar for the three populations (Fig. 2a, b). This shows that the DDI phenomenon is a public health burden in developed and developing nations regardless of access to medication or the type of healthcare system. Despite this, there are differences between the three populations that may be due to alternative factors. One is the well-documented differences in how computerized clinical decision systems display DDI alerts (passive or active) [35] or their ability to identify potential DDI (only 5% of the DDI alerts were common to the set of evaluated systems [36]) depending on the algorithms used [37]. The high variability in detecting DDI could mean that a specific drug pair may or may not be discouraged depending on the system used in each population.

Additionally, how patient care is organized directly impacts the prevalence of DDI, where continuity of care can significantly reduce the risk of DDI [38]. Alternatively, some discrepancies may be due to the type of healthcare system (public (Blumenau), public with co-payment (Catalonia), or private (Indianapolis)), or the availability of a primary care physician that could provide comprehensive care and increase attention to potential DDI. However, since our data does not include variables that would allow us to control explicitly for such factors, without more direct observational studies, we can only speculate about the roles of such factors in the DDI phenomenon. Notably, the lower prevalence of DDI in Catalonia and Indianapolis compared to Blumenau when considering the same period (18 months) may be partially because the former two populations have a greater number of drugs available (674 and 1088 for Catalonia and Indianapolis, respectively), some of which could be used to avoid DDI. However, it is essential to highlight that although there are several factors that may promote differences among the populations studied, in general terms, the three populations present substantially similar co-administration patterns.

Our statistical null model, designed to account for polypharmacy while preserving the same number of prescribed drugs and co-administrations per age, shows that the much higher prevalence of DDI in older age (in all populations) is not solely explained by the higher prevalence of co-administration in those age groups. Indeed, this worrisome result previously observed in Blumenau [10] is here shown to be even worse in Catalonia, where patients have a worse-than-random prevalence of DDI starting early in their twenties—reaching 2.7-fold higher-than-random prevalence for 55- to 59-year-olds (Fig. 2d). This worse-than-random prevalence of DDI remains even when separating men and women populations (Additional file 1: Fig. S6), questioning multimorbidity treatments and its current focus on geriatric patients.

Also similarly observed in all populations is a higher prevalence in women of both drug co-administration and interactions in comparison to men. The general prevalence of co-administration in women increases as they age. However, the largest difference from men occurs during peak reproductive age (age ranges 15–29; see Fig. 3 and Additional file 1: Fig. S3), which may be explained by women’s greater use of the healthcare systems during these years [39]. On the other hand, the gender imbalance in prevalence is generally much higher for interactions than for co-administrations (Additional file 1: Fig. S15). There are possible explanations as to why women have a generally higher prevalence of DDI. For instance, some drugs are women-specific, such as hormones and contraceptive drugs. Thus, women-specific drugs may partially explain the higher prevalence of DDI observed, particularly in younger women. The DDI pair ethinylestradiol and amoxicillin were jointly given to 0.98% of Catalan women but only to 0.0008% of men. In Blumenau, this same drug pair was given to 0.6% of women and no men. Unfortunately, we cannot infer from our data whether prescribers informed the patients of this DDI and the potential need for additional contraceptive methods during co-administration.

Additional reasons for the generally observed higher prevalence in women come from the fact that some diseases are more likely to affect women. For instance, osteoporosis is a skeletal disorder characterized by compromised bone strength [40] and is known to be diagnosed more frequently in women [41]. This gender-associated prevalence is observed in our data for the populations with disease diagnoses (Catalonia and Indianapolis, Additional file 1: Fig. S16). Bisphosphonates, such as alendronic acid, are used to treat osteoporosis, and, as a consequence, the prevalence of DDI related to alendronic acid is higher for women, especially those over 50. For instance, the *RRI* in women aged 60–64 between this drug and Ibuprofen is 1.8 and 1.34 in Catalonia and Blumenau, respectively. For men of the same age, this *RRI* is only 0.1 and 0.22 in both populations (Additional file 1: Table S9). The same can be seen in Indianapolis, albeit at a smaller scale. The *RRI* for alendronic acid and ibuprofen is only 0.04 for women in Indianapolis in the same 60–64 age range, and virtually no men administered this DDI in Indianapolis (Additional file 1: Table S9). This smaller *RRI* for Indianapolis is further supported by the comparatively small administration of alendronic acid (0.5% compared to 3.5% and 1.7%, see Additional file 1: Table S10), which likely stems from the decreased use of bisphosphonates in the US after the 2010 FDA bisphosphonate drug safety communication [42].

A deviation from the general trend of increased DDI prevalence in women is particularly noteworthy. In Indianapolis, men over 50 years of age do have a higher prevalence of DDI than women. Two factors drive this difference. First is the less frequent use of omeprazole in combination with benzodiazepines (widely used by women in the other two populations and correlated with significantly higher odds there (Additional file 1: Table S1)). Indeed, when we remove the omeprazole administration in Catalonia from our analysis (see the “Methods” section), men over 60 also show a higher prevalence of DDI than women (Additional file 1: Fig. S17). Second is the administration of some particular DDI that are given significantly more to men in Indianapolis, such as verapamil–midazolam, metoprolol–lidocaine, and lidocaine–carvedilol (see Fig. 5c). These observations highlight how our study also reveals specific gender-related differences in the DDI phenomenon for each population. With the tools we provide for further analysis, other researchers interested in this problem can further study and characterize specific DDIs of interest.

Another facet of the complex DDI phenomenon is patient multimorbidity. The proportion of patients with multimorbidities increases substantially with age, with almost 80% of the people suffering from at least two morbidities at the age of 65 [43]. As classical treatments are disease-independent, patients with multimorbidities are particularly at increased risk for DDI. For instance, patients with type 2 diabetes are known to be at higher risk for cardiovascular diseases and thrombotic complications [44]. Antidiabetic drugs such as glyburide, gliclazide, insulin lispro, and insulin glargine are often combined with NSAIDs such as ASA and anticoagulants such as acenocoumarol (the last being dispensed only in our Catalonia data) to treat both conditions, which increases the risk of hypoglycemia. Our work highlights that these are among the top 10 DDIs ranked by the number of patients they affect in all three populations. In addition, several of these drugs are usually co-administered for long periods, as characterized by our strength of interaction measure (Additional file 1: Table S1). Also related to anticoagulants, gout (an inflammatory disease characterized by elevated uric acid levels) increases the risk of thrombosis [45]. As a potential consequence, we find a higher-than-expected chance of concomitantly prescribing allopurinol with warfarin (Additional file 1: Table S11), a DDI that increases the risk of bleeding due to the potentiation of the anticoagulant effect [46]. Interestingly, the incidence of type 2 diabetes and gout are higher for men over 50 in Catalonia (Additional file 1: Fig. S16) and can potentially explain the higher administration of the DDIs mentioned above.

An essential aspect of our study is to exemplify how our large-scale study of the DDI phenomenon can lead to actionable interventions for public health benefit. For that purpose, we studied the role of the proton pump inhibitor (PPI) omeprazole on the observed DDIs in the three populations. PPI are the leading therapy for upper gastrointestinal disorders and prevention of gastric ulcers associated with the use of non-steroidal anti-inflammatories [47]. However, there is substantial evidence for inappropriate over-prescription of PPI, particularly of omeprazole [48–50]. For instance, in 2008, it was estimated that 100 million pounds from the National Health Service budget, and almost 2 billion pounds worldwide, were being spent unnecessarily on PPI [49]. Four-fifths of all PPI administrations in the UK were associated with omeprazole.

The lack of awareness, overuse, and misuse of PPI, together with the elevated number of drug interactions associated with omeprazole (phenytoin, methotrexate, and several benzodiazepine derivatives, among others), makes omeprazole one of the most significant culprits of DDIs. Indeed, in our study, omeprazole is the third and fourth most dispensed drug in Blumenau and Catalonia, respectively. Conversely, in Indianapolis, it is the 44th. Therefore, we simulated the substitution of omeprazole with alternative PPI—such as pantoprazole and lansoprazole––as a possible but actionable public health intervention. Such an intervention would reduce 20% of all men and 23% of all women currently administering a DDI in Catalonia (Additional file 1: Fig. S13b). This means 156,210 women and 92,533 men would be DDI-free in Catalonia if another PPI substituted their omeprazole prescription.

In contrast, extending the simulation to Indianapolis results in a much smaller reduction of DDI prevalence (only 2.5% fewer patients would not have been administered a DDI; see Additional file 1: Fig. S13c). This shows that in Indianapolis, the availability of PPI alternatives is being utilized to avoid known DDIs or ADRs involving this drug. Thus, as actionable interventions, our study suggests that Catalonia should encourage prescribing available PPI alternatives.

Given that a significant percentage of hospitalizations are due to drug–drug interactions, with ranges from 1.1 to 7.7% [4] depending on the type of study—prospective vs. retrospective—or the source of information and population analyzed, it is crucial to reduce DDI prevalence in the population. Special attention should be paid to the co-administration of major interactions, which have a prevalence between 3.06 and 5.34% in the populations analyzed. Indeed, the ASA-NSAIDs and digoxin–amiodarone co-administrations are among the DDI most frequently associated with hospital admissions and visits [4]. A study of adverse effects due to a DDI in France revealed that antithrombotic agents and antidepressants are the drugs most frequently implicated in ADRs resulting from a DDI (34% and 5%, respectively) [51]. Other studies have shown that DDIs involving drugs that reduce potassium levels (diuretics), centrally acting drugs (psychotropics), potassium-sparing drugs (angiotensin-converting enzyme), and antithrombotic agents comprised 80% of all potentially clinically significant DDIs [52]. All these drugs are in the top 100 most frequently co-administered DDIs in our study and can be retrieved from http://disease-perception.bsc.es/ddinteract/. To better understand which drug co-administrations lead to a higher risk of hospitalization, and the magnitude of this risk, it would be essential in the future to jointly analyze diagnoses and treatments. Since adverse effects generated by DDI are recorded in hospitals, this analysis will require the integration of data from hospitals and emergency rooms. In addition, in-situ studies focusing on the under-reporting of DDI—a very common phenomenon at different levels of healthcare [53–56]—should be included.

It is essential to note that DDI is not the only medication-related problem that can be prevented. For example, drugs used to treat a specific disease might negatively impact a comorbid condition, what is known as drug-disease interaction. Compared to drug–drug interactions, it has been reported that 16% of elderly patients in community dwellings suffer drug-disease interactions, compared to 25% of them taking interacting drugs [57]. These percentages rise to up to 64% of the patients in the primary-care setting [58], and 14% of the prescriptions generate alerts in clinical decision support systems [59]. As in the case of drug–drug interactions, the risk of disease-drug interactions also increases with age due to the increase in the number of co-occurring diseases and the number of drugs prescribed. For this reason, future work should focus on the joint study of disease diagnoses and drug administrations to measure the prevalence and impact of drug-disease interactions. Another medication-related problem is dosage problems, the most common type of medication error in pediatric patients [60] that should be analyzed in future works.

Some limitations of our study are warranted. First, we assume that the drugs dispensed were administered for their complete treatment length. In reality, patients may stop administration mid-treatment, and prescribers may substitute drugs for patients with complaints of adverse effects. Also, adverse drug reactions may, in some cases, be avoided by separating drug intake during the day (as is the case for levothyroxine and calcium [61], whose interaction could be avoided by separating the intake approximately 4 h) or adjusting dosage, constraining co-administration length or having the patient closely monitored depending on the context of the co-administration [62]. Thus, our results should be seen as a worst-case scenario for the administration of known DDIs. Nonetheless, since many still unknown DDIs certainly exist and our analysis only covers DDIs known in 2011 (see the “Methods” section), the true importance of the DDI phenomenon is likely larger than what we observed. In addition, the relatively short study periods for Blumenau and Indianapolis compared to Catalonia may mask shifts in drug availability policy. This certainly highlights the importance of pursuing future studies with longer periods of observation as data becomes available. In addition, ICD codes may sometimes not perfectly correspond to the diagnosis documented by physicians, and there may be slight variations in the prevalence of certain diseases. Despite this, ICD-10 have been used in a multitude of published articles focused on analyzing comorbidity relationships [63–66], including the ones analyzed here for Catalonia [67–69].

Finally, it is necessary to consider that, since the number of therapeutic targets is limited [70], sometimes there may not be an alternative to avoid drug interaction. For example, patients with heart failure take furosemide and digoxin, one of the significantly co-administered drug–drug interactions in all 3 study populations (Additional file 1: Table S11), the intake of which significantly increases the risk of hospitalization for digoxin intoxication [30]. Unfortunately, on many occasions, both drugs have to be co-administered to patients with heart failure, as they are used to treat different aspects of the disease: furosemide (and diuretics in general) is recommended for patients with symptomatic heart failure to control pulmonary congestion and peripheral edema, and digoxin is taken to enhance cardiac contractility, improve baroreceptor function, and decrease sympathetic tone. It is important to make a risk/benefit balance, as in the case of combined antiplatelet and anticoagulant therapy. For example, the combination of both types of drugs has been shown to provide additional benefits over the use of anticoagulants alone in patients with some diseases such as prosthetic heart valves [71]. Similarly, the combination of heparin and aspirin during the course of pregnancy can increase the birth rate in women with antiphospholipid antibodies [72], demonstrating that the context in which both drugs are given is important.

## Conclusions

Our large-scale epidemiological analysis shows that DDIs are indeed a problem that affects a substantial proportion of patients in the three distinct populations studied. Ours is the first study to compare the DDI phenomenon in three large and distinct public and private healthcare systems and follow close to 6 million patients for over a decade. Because we studied very diverse populations and health systems, from developing to developed countries, our results are likely generalizable to other nations where access to EHR data is still difficult or non-existent. Of particular importance is that similar gender and age differences exist in the administration of known DDIs in all observed public health systems, albeit with some context-specific differences we also characterize. Thus, physicians, drug developers, and healthcare professionals should be aware that the existence of gender and age differences need to be taken into consideration in drug management. The analysis, results, and tools we provide can be used by others to investigate additional actionable interventions. Indeed, our study emphasizes that much more attention should be put into understanding and reducing the DDI phenomenon and its biases. Because interactions between cultural, economic, and biological factors are likely at play, in addition to computational and epidemiological studies such as ours, the DDI phenomenon calls for greater interdisciplinary collaboration. We hope that by uncovering such a large footprint of the DDI phenomenon, with the burden it represents to patients and healthcare systems alike, we also contribute to awareness of the need to accelerate disruptive drug research toward new and safer therapeutic targets, particularly for chronic conditions.

### Supplementary Information

**Additional file 1: Table S1**. Top 10 most co-administered DDI. **Table S2**. Percentage of patients taking omeprazole. **Figure S1**. Diagrams of drugs and DDI co-administered in the three populations. **Table S3**. Relative risk of DDI co-administration. **Table S4** and **S5**. Odds Ratio of co-administering drugs (*S4*) and DDIs (*S5*) as a function of the population studied. **Table S6**. DDI significantly co-administered more than the expected by chance in the three populations. **Figure S2**. Percentage of patients co-administered 2 or more drugs. **Figure S3**. Prevalence of drug and DDI co-administration. **Figure S4**. Evolution of ethinylestradiol administrations from 2008 to 2018. **Figure S5**. Prevalence of drug and DDI co-administration after removing ethinylestradiol. **Figure S6**. Prevalence of DDI by gender in the null model. **Figure S7 and S8**. Prevalence of DDI co-administration by severity during the first 18 months (*S7*) or the entire study period (*S8*). **Figure S9, S10, and S11**. DDI Networks from Blumenau (*S9*), Catalonia (*S10*) and Indianapolis (S11). **Table S7**. Major DDI with discordant gender-associated prevalence. **Figure S12**. Gender associated differences in co-administration with aging. **Table S8**. Odds Ratio of administering different proton pump inhibitors in combination with diazepam and clonazepam. **Figure S13**. DDI prevalence before and after replace omeprazole with other PPI. **Figure S14**. As *Figure S13* but by severity. **Figure S15**. Gender-associated relative risk of drug and DDI co-administration. **Figure S16**. Number of type II diabetes, gout, and osteoporosis diagnoses in Catalonia and Indianapolis. **Table S9**. Gender-associated relative risk of alendronic acid – ibuprofen co-administration. **Table S10**. Percentage of patients administered bisphosphonates. **Figure S17**. DDI prevalence after removing Omeprazol-associated interactions. **Table S11**. Strength of co-adminstration of top 12 significantly co-administered DDI.

## Data Availability

Data is available at: https://github.com/rionbr/DDI-Cat-Indy-Bnu.

## Abbreviations

ADR: Adverse drug reactions
ATC: Anatomical Therapeutic Chemical
CHI: Catalan Health Institute
DDI: Drug–drug interaction
EHR: Electronic health records
HIS: Health information systems
PC: Prevalence of co-administration
PI: Prevalence of interaction
PPI: Proton pump inhibitors
RRC: Relative risk of co-administration
RRI: Relative risk of interaction
