# Supplementary material for: Prevalence and differences in the co-administration of drugs known to interact: an analysis of three distinct and large populations
Source: BMC Med. 2024 Apr 19;22:166. doi: 10.1186/s12916-024-03384-1 (PMC11027217; doi:10.1186/s12916-024-03384-1)
Supplement: Supplementary file 1 — Additional file 1: Table S1. Top 10 most co-administered DDI. Table S2. Percentage of patients taking omeprazole. Figure S1. Diagrams of drugs and DDI co-administered in the three populations. Table S3. Relative risk of DDI co-administration. Table S4 and S5. Odds Ratio of co-administering drugs (S4) and DDIs (S5) as a function of the population studied. Table S6. DDI significantly co-administered more than the expected by chance in the three populations. Figure S2. Percentage of patients co-administered 2 or more drugs. Figure S3. Prevalence of drug and DDI co-administration. Figure S4. Evolution of ethinylestradiol administrations from 2008 to 2018. Figure S5. Prevalence of drug and DDI co-administration after removing ethinylestradiol. Figure S6. Prevalence of DDI by gender in the null model. Figure S7 and S8. Prevalence of DDI co-administration by severity during the first 18 months (S7) or the entire study period (S8). Figure S9, S10, and S11. DDI Networks from Blumenau (S9), Catalonia (S10) and Indianapolis (S11). Table S7. Major DDI with discordant gender-associated prevalence. Figure S12. Gender associated differences in co-administration with aging. Table S8. Odds Ratio of administering different proton pump inhibitors in combination with diazepam and clonazepam. Figure S13. DDI prevalence before and after replace omeprazole with other PPI. Figure S14. As Figure S13 but by severity. Figure S15. Gender-associated relative risk of drug and DDI co-administration. Figure S16. Number of type II diabetes, gout, and osteoporosis diagnoses in Catalonia and Indianapolis. Table S9. Gender-associated relative risk of alendronic acid – ibuprofen co-administration. Table S10. Percentage of patients administered bisphosphonates. Figure S17. DDI prevalence after removing Omeprazol-associated interactions. Table S11. Strength of co-adminstration of top 12 significantly co-administered DDI. [file 12916_2024_3384_MOESM1_ESM.docx]

# Supplemental Material

Prevalence and differences in the co-administration of
drugs known to interact: an analysis of three distinct and large populations

Jon Sánchez-Valle^1*^*^†^*, Rion Brattig Correia^2*^*^†^*, Marta Camacho-Artacho, Rosalba Lepore^1,3^, Mauro M. Mattos^4^, Luis M. Rocha^2,5*^ and Alfonso Valencia^1,6*^

## **S1 Additional results on observed drug-drug interactions**

To study the details of the DDI phenomenon in each population, here we present results using the entire temporal data: 18 months for Blumenau, 11 years for Catalonia, and 2 years for Indianapolis. We first observe that 211, 1,782, and 1,483 unique DDI were administered in the Blumenau, Catalonia, and Indianapolis populations, respectively. The top DDIs (and their severity) for each population are shown in table S1, and the entire list is available at [http://disease-perception.bsc.es/ddinteract/.](http://disease-perception.bsc.es/ddinteract/)

The concomitant use of Omeprazole with various benzodiazepines (Clonazepam, Diazepam, and Alprazolam) is among the most frequent DDIs in Blumenau and Catalonia and is associated with higher prevalence in women in both cities. In Indianapolis, in contrast, the most frequent DDIs are the concomitant use of: two anticoagulants (Acetylsalicylic acid (ASA) Heparin), an anticoagulant and insulin (ASA combined with Insulin lispro or Insulin glargine), and a beta-blocker and a local anesthetic (Metoprolol and Lidocaine), all of which are associated with greater prevalence (*PI*) in men.

Drug availability differs in the three health care systems. Considering only the common set of 106 drugs dispensed in all three populations (fig. S1a), Catalonia is the population with the largest number of administered unique DDIs (206), followed by Indianapolis (183), and Blumenau (175), as shown in see fig. S1b. In addition, five DDIs are only observed in Catalonia—all associated with epilepsy treatment. Three of the interactions involve Phenytoin (co-administered with Disulfiram, Betamethasone and Mebendazole) and the two others involve Phenobarbital (co-administered with Betamethasone and Nifedipine). Interestingly, three of the four DDIs only observed in Blumenau and Indianapolis also involve epilepsy-treatment drugs in different combinations from Catalonia: Valproic acid (co-administered with Erythromycin, Phenytoin, and Phenobarbital).

**S2 Strength of the co-administration**

Table S11 shows the DDIs that are co-administered more than expected by change and are common in the three populations, ranked based on the strength of their association and the number of patients affected by them. Half of these interactions pose a major risk for health, leading to hyperkalemia and kidney failure (Spironolactone and Losartan), increased risk of bleeding (Warfarin and Amiodarone), and excess mortality (Digoxin and Amiodarone) [29].

The highest ranked DDI pair is Digoxin (cardiac glycoside) with Furosemide (diuretic) combination, which significantly increases the risk of hospitalization for Digoxin intoxication [30]. In addition to Furosemide, Digoxin is significantly associated also with two drugs used to treat high blood pressure associated to heart failure (Spironolactone and Carvedilol), an antiarrhythmic drug (Amiodarone), and Levothyroxine, a drug used to treat thyroid hormone deficiency. It has been shown that, even at subclinical levels, hypothyroidism worsens heart failure prognosis [31].

## **S3 Drug-drug interaction networks**

Both Blumenau and Catalonia have larger number of distinct DDIs being co-administered to a higher proportion of women (see number of red edges in networks, such as fig. 4). In Blumenau, out of the 211 DDI observed 142 (67%) have an higher prevalence in women while only 69 (33%) have a higher prevalence in men. For Catalonia these numbers are 981 (55%) and 801 (45%), for women and men, respectively. For Indianapolis, even though the populationlevel prevalence of DDI is higher for women (as seen in table 1), there is almost the same proportion of distinct DDIs associated with a prevalence in both genders, 729 (49.8%) in women and 744 (50.2%) in men. Interestingly, when only DDIs co-administered more than expected by chance are considered (Fisher’s exact test, FDR ≤ 0.05), the proportions of gender-based relative risk DDIs are reversed in Blumenau and Catalonia. In Blumenau, out of the 48 significant DDI observed, 23 (48%) have a higher prevalence in women and 25 (52%) have a higher prevalence in men, a 67 − 48 = 19% decrease for women. For Catalonia these same numbers are 103 (42%) and 143 (58%), for women and men, respectively, a 13% decrease for women. And lastly for Indianapolis the proportions are 83 (38.6%) significant DDIs for women and 132 (61.4%) for men, also a decrease of 11.1% to women. Naturally, a significant DDI proportion reduction for women denote an increase for men.

## **S4 – Supplementary Methods (equations)**

**Patients:**

*u* ∈ *U*

**Drugs:**

*i,j* ∈ *D*

**Subset of patients dispensed drug i:**

*U_i_* ∈ *U*

**Subset of drugs dispensed to patient *u:***

*D^u^* ⊆ *D*

**Set of distinct administration intervals *a^i,u^_n_* of drug *i* to patient *u***:

$$A_{i}^{u}\equiv\{a^{i,u}\}$$

**Total number of administrations**:

$${}_{i}^{u}=|A_{i}^{u}|$$

**Time units a patient *u* is administered a drug *i*:**

${}_{i}^{u}=\sum a^{i,u}$.

**Patient *u* co-administered drug pair (*i,j*):**

${}_{i,j}^{u}\in\{0,1\}$ ; ${}_{i,j}^{u}=({}_{i,j}^{u}>0)$

**Co-administration of drugs (*i,j*) known to interact to patient *u*:**

${}_{i,j}^{u}\in\left\{ 0,1 \right\}$ ; ${}_{i,j}^{u}=({}_{i,j}^{u}=1\wedge{}_{i,j}=1)$

**Probability co-administering drug pair (i,j) when administered drug *i*:**

$${}_{i,j}=\frac{|U_{i,j}|}{U_{i}}$$

*this measure is not symmetrical* $({}_{i,j}\neq{}_{j,i})$.

**Strength of co-administration of drug pairs to a patient *u*:**

$${}_{i,j}^{u}=\frac{{}_{i,j}^{u}}{({}_{i}^{u}+{}_{j}^{u}-{}_{i,j}^{u})}$$

**Mean value for the cohort of patients who administered drug pair (*i,j*) concomitantly:**

${}_{i,j}=\frac{\sum_{u\in U_{i,j}} {}_{i,j}^{u}}{|U_{i,j}|}$

**Strength of a DDI:**

$${}_{i,j}={}_{i,j}x{}_{i,j}$$

**Conditional likelihood of drug interaction:**

$${}_{i,j}={}_{i,j}x{}_{i,j}$$

**Prevalence of co-administration (PC):**

$$\left| U \right|/|U|$$

**Prevalence of interaction (PI):**

$$\left| U \right|/|U|$$

**Relative risk of co-administration for women:**

$${RRC}^{W}=\frac{P({}^{u}>0|u\in U^{W})}{P({}^{u}>0|u\in U^{M})}=\frac{|U^{,W}|/|U^{W}|}{|U^{,M}|/|U^{M}|}$$

**Relative risk of co-administration for men:**

$${RRC}^{M}=1/{RRC}^{W}$$

**Relative risk of interaction for women:**

$${RRI}^{W}=\frac{P({}^{u}>0|u\in U^{W})}{P({}^{u}>0|u\in U^{M})}=\frac{|U^{,W}|/|U^{W}|}{|U^{,M}|/|U^{M}|}$$

**Relative risk of interaction for men:**

$${RRI}^{M}=1/{RRI}^{W}$$

**Prevalence of co-administration for age group** $\left[ \boldsymbol{y}\boldsymbol{1,y}\boldsymbol{2} \right]$**:**

$${PC}^{[y1,y2]}=\frac{|U^{,[y1,y2]}|}{|U^{[y1,y2]}|}$$

**Prevalence of interaction for age group** $\left[ \boldsymbol{y}\boldsymbol{1,y}\boldsymbol{2} \right]$**:**

$${PI}^{[y1,y2]}=\frac{|U^{,[y1,y2]}|}{|U^{[y1,y2]}|}$$

**Prevalence of interaction by gender (**$\boldsymbol{g\in\{W,M\}}$**):**

${PI}^{\left[ y1,y2 \right],g}$

**Prevalence of interaction by gender (**$\boldsymbol{g\in\{W,M\}}$**) and drug pair (i,j):**

${PI}_{i,j}^{\left[ y1,y2 \right],g}$

**Relative risks constrained on age ranges, gender, and drug pairs:**

${RRI}^{\left[ y1,y2 \right],W}$ and ${RRI}_{i,j}^{\left[ y1,y2 \right],W}$

**Number of patients who administered a DDI involving drug *i*:**

$$|U_{i}|$$

**Probability of patients who administered drug *i* to be exposed to a DDI associated with that drug:**

$$P\left( U_{i} \right)=\frac{|U_{i}|}{|U^{i}|}$$

**Expected prevalence of interactions after randomly administering drugs:**

$$\hat{PI}^{[y1,y2]}$$

**Relative risk of interaction (observed vs. null model):**

$$RR={PI}^{\left[ y1,y2 \right]}/\hat{PI}^{[y1,y2]}$$

**Table S1.** Top 10 DDI observed in the three populations ranked based on the number of patients $(|U_{i,j}|)$ taking them. ${}_{i,j}$ denotes the strength of interaction. ${RRI}^{W}$ denotes the relative risk of interaction for women, with values larger than (smaller than) 1 denoting higher risk for women (men). The false discovery rate (FDR) and odds ratio represent the significance of the interaction, i.e. whether both drugs are administered more than expected by chance. DDI severity shown as retrieved from Drugs.com [28].

**Table S2.** Number and percentage of patients who administered Omeprazole in Blumenau, Catalonia, and Indianapolis.

**Figure S1.** Numbers of drugs administered and drug-drug interactions in Blumenau, Catalonia, and Indianapolis. (a) Venn diagram representing the number of unique drugs dispensed in the three populations. (b) Venn diagram representing the number of unique drug-drug interactions in Blumenau, Catalonia and Indianapolis, when considering exclusively the 106 drugs administered in the three populations. (c) Spearman’s correlations of the strength of interaction for all 149 DDI pairs observed in the shared set.

**Table S3.** Relative risk for women $(RRW)$ of drug co-administration (RRC) and interactions (RRI); the latter is also computed for types of interactions as per drugs.com (minor, moderate, and major). The percentage of patients of each gender (M = man; W= woman) for each case is also shown. Values shown for all three populations for the whole study period, that is, 18 month for Blumenau, 11 years for Catalonia, and 2 years for Indianapolis.

**Table S4.** Odds Ratio (OR) of co-administering drugs as a function of the population studied both for the general population and separately for women and men during the first 18 months of the study. 95% confidence intervals are indicated in parentheses.

**Table S5.** Odds Ratio (OR) of co-administering interacting drugs as a function of the population studied both for the general population and separately for women and men during the first 18 months of the study. 95% confidence intervals are indicated in parentheses.

**Table S6.** Drug-drug interactions co-administered significantly more than expected were 246 out of 1,782 in Catalonia, 5 out of 1,483 in Indianapolis, and 48 out of 211 in Blumenau. The 12 shown in the table are those common to the three populations, ranked by population “footprint” (${}_{i,j}$ and ${}_{j,i}$ values). Values in bold denote the higher value between drugs *i* and *j*. Strength of drug interaction is shown for each population. To allow cross population comparison, the number of patients is shown as a percentage of the overall patient population ($|U_{i,j}|/|U|$).

**Figure S2.** Percentage of patients co-administered 2 or more drugs simultaneously in Blumenau, Catalonia, and Indianapolis.

**Figure S3.** Prevalence of co-administration of drugs (a-c) and drugs known to interact (d-f) by age and gender in Blumenau, Catalonia, and Indianapolis analyzing the whole study period. Red and blue colors denote the prevalence of co-administration in women and men respectively.

**Figure S4.** Evolution of the number of (a) administrations of Ethinylestradiol and (b) co-administrations of Ethinylestradiol and Amoxicillin from 2008 to 2018 in Catalonia. In b, red and blue colors denote the number of co-administrations in women and men respectively.

**Figure S5.** Prevalence of co-administration and interaction by age group and gender in Catalonia every two years after the removal of Ethinylestradiol dispensations. Red and blue colors denote the prevalence of co-administration in women and men, respectively. Relative risk of co-administration and interaction for women per age group shown above the points.

**Figure S6.** Prevalence of interaction and its associated null model by age group and gender in Blumenau, Catalonia, and Indianapolis during the first 18 months of administrations. Circles denote the values obtained with the real data, while asterisks denote the values obtained using the null model. Risk for women (*g* = *W*) shown in red (a-c) and men (*g* = *M*) shown in blue (d-f), respectively. The associated relative risk is shown over the points for each age group.

**Figure S7.** Prevalence of interaction by severity in Blumenau (a-d), Catalonia (e-h), and Indianapolis (i-l) during the first 18 months of administrations. Red and blue colors denote the prevalence of co-administration in women and men respectively. The classification as minor (a, e, i), moderate (b, f, j), and major (c, g, k) interactions are extracted from drugs.com[28]. Those interactions with no description of the severity are denoted as “unknown severity interactions” (d, h, l). Relative risks of interaction for women per age group is shown above points. Asterisks denote significant differences (Fisher’s exact test).

**Figure S8.** Prevalence of interaction by severity in Blumenau (a-d), Catalonia (e-h), and Indianapolis (i-l) during the whole study period for each population. Red and blue colors denote the prevalence of co-administration in women and men respectively. The classification as minor (a, e, i), moderate (b, f, j), and major (c, g, k) interactions are extracted from drugs.com[28]. Those interactions with no description of the severity are denoted as “unknown severity interactions” (d, h, l). Relative risks of interaction for women per age group is shown above points. Asterisks denote significant differences (Fisher’s exact test).

**Figure S9.** Blumenau DDI Network. Nodes denote drugs *i* involved in at least one co-administration known to be a DDI. Node color represents the highest level of primary action class, as retrieved from Drugs.com (see legend in fig. 3). Node size represents the probability of patients to be affected by a DDI involving the drug $P(U_{i})$. Edge weights are the values of ${}_{i,j}$. Edge colors denote ${RRI}_{i,j}^{g}$, where $g\in M,W$, to identify DDI edges that are higher risk for women (red) or men (blue). Color intensity for ${RRI}_{i,j}^{g}$ varies in [1,5]; that is, values are clipped at 5.

**Figure S10.** Catalonia DDI Network. Nodes denote drugs *i* involved in at least one co-administration known to be a DDI. Node color represents the highest level of primary action class, as retrieved from Drugs.com (see legend in fig. 3). Node size represents the probability of patients to be affected by a DDI involving the drug $P(U_{i})$. Edge weights are the values of ${}_{i,j}$. Edge colors denote ${RRI}_{i,j}^{g}$, where $g\in M,W$, to identify DDI edges that are higher risk for women (red) or men (blue). Color intensity for ${RRI}_{i,j}^{g}$ varies in [1,5]; that is, values are clipped at 5.

**Figure S11.** Indianapolis DDI Network. Nodes denote drugs *i* involved in at least one co-administration known to be a DDI. Node color represents the highest level of primary action class, as retrieved from Drugs.com (see legend in fig. 3). Node size represents the probability of patients to be affected by a DDI involving the drug $P(U_{i})$. Edge weights are the values of ${}_{i,j}$. Edge colors denote ${RRI}_{i,j}^{g}$, where $g\in M,W$, to identify DDI edges that are higher risk for women (red) or men (blue). Color intensity for ${RRI}_{i,j}^{g}$ varies in [1,5]; that is, values are clipped at 5.

**Table S7.** Major DDI co-administered in the three populations with discordant gender-associated prevalence (interactions that are associated with a higher prevalence in women in one or two populations and a higher prevalence in men in the other). For instance, column “(Cat = Indy) ≠ Bnu” list major DDI with shared gender-associated prevalence in Catalonia and Indianapolis but reversed gender prevalence in Blumenau. Similarly, the same logic describes the other columns.

**Figure S12.** Gender-associated differential co-administration of the drug-drug interaction co-administration in the three populations with ageing. Heatmaps’ colors denote the increased percentage of women (red) and men (blue) taking each DDI at each age bin in the different populations, calculated as ${PI}_{i,j}^{\left[ y1,y2 \right],W}-{PI}_{i,j}^{\left[ y1,y2 \right],M}$.

**Table S8.** Fisher’s exact test results analyzing the significance of the association between omeprazole and diazepam/clonazepam compared to all the other proton pump inhibitors in Catalonia and Indianapolis. Arrows (← and →) denote the direction of the odds-ratio and the p-values, where arrows pointing to the right denote a higher risk for omeprazole, while arrows pointing to the left denote a higher risk for the alternative proton pump inhibitors.

**Figure S13.** Prevalence of interaction before and after replacing Omeprazole with other protonpump inhibitors (PPI).

**Figure S14.** Prevalence of interaction by severity score before and after replacing Omeprazole with other PPIs.

**Figure S15.** Relative risks of co-administration and interaction for women per age group for Blumenau, Catalonia, and Indianapolis in the first 18 months of administrations. Asterisks denote significant differences (Fisher’s exact test). Note scales are different among plots.

**Figure S16.** Type II diabetes, gout, and osteoporosis diagnoses in Catalonia and Indianapolis for women (red) and men (blue).

**Table S9.** Relative risk of interaction for women, and prevalence of interaction for both women $({PI}_{i,j}^{\left[ y1,y2 \right],W})$ and men $({PI}_{i,j}^{\left[ y1,y2 \right],M})$ for the DDI between Alendronic acid and Ibuprofen by age range and gender.

**Table S10.** Number and percentage of patients who administered Alendronic acid, Ibandronate, Risedronatem, and Zoledronic acid in Blumenau, Catalonia, and Indianapolis during the entire study period.

**Figure S17.** Prevalence of interaction by age and gender in Catalonia after the removal of Omeprazole associated interactions. Red and blue colors denote the prevalence of co-administration in women and men respectively. Relative risks of interaction for women per age group displayed above the points. Asterisks denote significant differences (Fisher’s exact test).

**Table S11.** The 12 drug-drug interactions administered more than expected and common to the Blumenau, Indianapolis, and Catalonia populations, ordered based on the rank product of the strength of the association $({}_{i,j})$ and the number of patients (%) in the three populations. To allow cross population comparison, number of patients are shown as a percentage of the overall patient population $(|U_{i,j}|/|U|)$.
